# The Country Doctor

**Published:** 1900-07

**Authors:** W. E. Fowler

**Affiliations:** Huntsville, Texas


For the Texas Medical Journal.

### The Country Doctor.

BY W. E. FOWLER, M. D., HUNTSVILLE, TEXAS.

I admire to the fullest extent the ennobling energy and Christian charity upon which the great city hospitals are founded; I keenly appreciate and heartily applaud the high order of medical and surgical acumen that have made them famous; and I cheerfully give a full measure of credit to the learned and gifted specialists in charge of the various subdivisions of these invaluable institutions; but, at the same time, I believe that the country doctors are contributing entirely too much revenue to their support. This is not, however, the direct fault of the specialists and institutions referred to; it is, rather, the evidence or exhibition of a woeful lack of self-confidence on the part of the physicians whose fields of labor are far removed from the chief centers of medical learning, and to whom the majority of the country's population must look for succor when the cold hand of disease or the heavy stroke of accident falls upon them.

The ordinary physician may not advertise beyond a plain and unostentatious announcement of his professional calling, while it is entirely within the safety line vouchsafed by the code of ethics for the hospitals to proclaim their wares with a flourish of trumpets. At any rate, such is the case, as any one familiar with the columns of the daily press can testify. And then these medical men go into the journals with wonderful accounts of still more wonderful cures and operations that daily occur within their aseptic wards—painted with the glowing colors snatched from the rainbow where it dips to the earth, and which they have easily reached—until we poor little fellows away off here in the obscure nooks of the world feel our insignificance to an extent that almost induces us to turn away from our chosen profession; and there are times, perhaps, when we would do this very thing but for two good reasons, viz.: Because it is impossible, owing to financial conditions; and because, we conclude the obstacles to our further progress, so vividly portrayed by these erudite and fluent writers, are probably not, after all, so wholly insurmountable as they would have us believe. But they have, nevertheless, completely destroyed the self-reliance (which is the *sine qua non* of success) of many good men, and thereby quenched forever lights that, otherwise, might have illuminated many dark corners of the medical world.

We read many of their articles with a feeling of shuddering awe and trembling wonderment; but we admire their genius and skill; their unerring diagnosis and boldness in the execution of difficult operations devised by themselves often amazes and astonishes us. They are careful to tell us in minutest detail of all the *essentials* to be observed and practiced in order to the achievement of brilliant success; but, by their prolix *essentials,* they—as it often appears to me—endeavor to convince us that it is about next to impossible for the country doctor to do more than lance a boil and diagnose a case of intermittent fever! So, therefore, it should not seem strange if many of us arrived at the sophistical conclusion that no sort of operations should be undertaken outside of a hospital with tiled floors, glass tables and trained nurses; and, being thus deceived as to our own opportunities and resources, most of our surgical cases are sent to the cities to furnish bouquets to adorn the reputations of men in nowise our superiors. Most of these cases could just as well be treated at home; and I am glad that the voices of other country physicians are being heard to echo this same sentiment.

I would not be understood as crying down the hospitals, or as under-estimating the valuable work of the eminent medical gentlemen connected with them; but I certainly think it very unfortunate for the poor people of the country, as well as detrimental to the country's doctors, that they have been permitted to form a trust—so to speak—and corner the surgery of all the outlying districts. I give them full credit for the vast volumes of information and learning which they are constantly accumulating and sending out, but I contend that the flourish of the hospital writers becomes a species of advertisement powerful in filling their wards and coffers at the expense of the country physicians. And this is done in great part by simply over-awing the timid, causing them to send away their patients instead of treating them at home; and in concluding this view of my subject, I would urge the earnest co-operation of the country members of the profession in a heroic effort to correct this evil.

I believe in clean surgery. I believe that no operation—no matter how insignificant—should be performed, except under the strictest antiseptic conditions; but I believe this can be done without tiled floors, glass tables and trained nurses—without a systematic scrubbing of hands and arms in four or five bowls filled with as many cleansing and germ-killing solutions. One can find, at all times, an abundance of soap, water, carbolic acid and bichloride solutions; and, if these be properly used, I don't think there should linger a fear of septicæmia. And when surgical instruments are plunged into hot carbolized water and permitted to remain submerged for a few moments, I am very sure that any microbes that might have been roosting on their surfaces will be found *hors du combat,* and therefore in a state of complete "innocuous desuetude." But a knowledge of the practical facts set out in this contention must come through actual experience and personal observation—not through the published clinical lectures of our hospital brethren of the large cities. Many good and promising men, however, are often disheartened and driven to obscure and helpless poverty before the opportunities of such convincing and man-making experience are offered and their rescue effected. For example: We read the complicated technique of a laparotomy, as delineated by the celebrated hospital men; and, if we didn't know better, we would most likely arrive at the conclusion that it could not be performed with any possible degree of success or safety outside of a hospital elaborately furnished with a hundred costly and wonderful modern devices which we never saw, and never expect to see. But, if we are not completely over-awed and dispirited at the very outset, we are likely to sit down and muse about as follows: "I think my medical knowledge is fairly good; my opportunities at college were such as to give me a good start on the road to success, and I have never ceased to put forth my best efforts; my knowledge of anatomy and pathological conditions is at least equal to that of many successful men; I am reasonably familiar with the technique of the text-book operations; I know the immediate effect of this or that cut; I am very well acquainted with the various germ theories and the use of antiseptics; my work so far has been satisfactory; I know, also, that the private home is scarcely ever the abiding place of disease-producing germs, while the hospitals more or less teem with them; I guess I shall continue to do my own work!" And right here I wish to assert my belief that the country doctor who thus reasons will extend his work and succeed; and he will not only succeed and build up for himself an enviable reputation, but he will ameliorate much suffering, correct many evils and prolong many lives. He will paint the fresh rose of health on many cheeks already pale and shrunken by the suffocating breath of approaching death; and for this reason every physician should be as brave and courageous as the soldier that storms strongholds and captures bellowing batteries.

Now, as to laparotomies. Not long ago a man was struck with a long knife, by which a very extensive "laparotomy" was performed upon him without the slightest preparation or pretense at aseptic conditions! The point of the knife entered at the umbilicus and, extending inward, upward and outward, ripped open the right side of the abdomen from the point of entrance to the ninth rib, disemboweling the man. Neither his clothing nor his person was in an aseptic condition. Gathering up his protruding bowel in his hands, the wounded man walked over one hundred yards before receiving any assistance. I saw him in a very few moments. I found the intestine covered with dirt and beginning to puff up with accumulating gas. According to the lectures of some of our eloquent hospital surgeons, this man would have died if operated upon under such unfavorable conditions. But he smiled and refused to do anything of the kind. He was placed upon an operating table, his clothing cut away and the parts about the wound cleansed with warm carbolized water. Then the protruding links of intestine were similarly treated and made aseptic. After thoroughly examining the intestine and ascertaining that it had not been cut, it was carefully replaced and the wound closed with silk—the figure-of-eight stitch being employed. The wound was then covered with iodoform gauze, absorbent cotton and bandage. On the sixth day the dressing and sutures were removed. There was complete union by first intention. Yes, the man was well; but a broad bandage of support was worn for a short time as a precautionary measure. Such cases as this serve to strengthen the failing self-confidence of the country physician—who must also be a surgeon.

Another man was slashed to pieces with a razor in the hands of a maniac. One of the cuts started at the spinal column and, following the sixth rib, extended to the sternum—severing everything down to the bone. It was a most ghastly sight. Numerous other gaping cuts were upon the body—their aggregate length being five feet and ten inches. He lost much blood. As soon as the bleeding points could be controlled, the wounds were closed—the muscles with catgut, the surface with silk. This man made a rapid and uneventful recovery, all the wounds healing by first intention, and thus proving to the timid doctor that a little self-reliance would enable him to do much that he shuns. If these severe accidents can be so easily managed, why should we hesitate to operate when occasion requires?

I could cite numerous cases similar to the above, but, as my paper is long enough, I shall refrain from so doing. I take it that the successful management of such grave accidents should teach valuable lessons—not alone for the benefit of physicians, but for the public as well. The time has come when most people believe that when the knife is to be used it must be done in some city hospital. If Col. Doe has a case of piles—or even an insignificant clot—he must jump aboard of a train and go to the city for treatment; and if Maj. Roe has a stricture of the urethra he does likewise, when the family physician could treat them quite as intelligently and successfully, and with just as little pain. And just here I wish to assert that the family physician would treat them far more considerately than the city man; because, with the latter, it is little more than a matter of dollars and cents. But with the former, there is a great deal more than a fee involved—there are social and neighborly ties and considerations. The people should be educated to a full appreciation of this fact. It would be worth many dollars to them, and would keep them in their own homes, to be cared for by loved ones instead of lying in hospital wards, depending upon the hired assistance of strangers for necessary attention. Yes, the family physician should be the subject of a greater trust on the part of those he so faithfully serves, and to whom he gives genuine sympathy as well as professional care, than is sometimes—a great many times—the case; and it would be far better for all concerned if this deplorable condition did not exist.

